# Tackling resistance: emerging antimalarials and new parasite targets in the era of elimination

**DOI:** 10.12688/f1000research.14874.1

**Published:** 2018-08-01

**Authors:** Emily S. Mathews, Audrey R. Odom John

**Affiliations:** 1 Department of Pediatrics, Washington University School of Medicine, St. Louis, Missouri, USA; 2 Department of Molecular Microbiology, Washington University School of Medicine, St. Louis, Missouri, USA

**Keywords:** Plasmodium, antimalarial, drug targets, drug discovery, resistance

## Abstract

Malaria remains a significant contributor to global human mortality, and roughly half the world’s population is at risk for infection with *Plasmodium *spp. parasites. Aggressive control measures have reduced the global prevalence of malaria significantly over the past decade. However, resistance to available antimalarials continues to spread, including resistance to the widely used artemisinin-based combination therapies. Novel antimalarial compounds and therapeutic targets are greatly needed. This review will briefly discuss several promising current antimalarial development projects, including artefenomel, ferroquine, cipargamin, SJ733, KAF156, MMV048, and tafenoquine. In addition, we describe recent large-scale genetic and resistance screens that have been instrumental in target discovery. Finally, we highlight new antimalarial targets, which include essential transporters and proteases. These emerging antimalarial compounds and therapeutic targets have the potential to overcome multi-drug resistance in ongoing efforts toward malaria elimination.

## Introduction

Malaria has posed a risk to human life since the origin of our species. Despite this long history, it was not until 1880 that French army surgeon Charles Louis Alphonse Laveran discovered intraerythrocytic parasites in the blood of a patient with malaria ^1^. Immediately following Laveran’s discovery, crucial aspects of this infection, including species classification and the details of the human–mosquito transmission cycle, were revealed ^2– 6^. These early studies shaped our understanding of the protozoan parasites of genus *Plasmodium* that cause malaria. In the complex life-cycle of *Plasmodium* spp., human infection begins with the bloodmeal of a female *Anopheles* mosquito. Parasites migrate to the liver, where they undergo a large, asymptomatic expansion, emerging to invade red blood cells and initiate asexual replication. A small fraction of these blood-stage parasites terminally differentiate into gametocytes that are taken up by the mosquito to complete sexual replication and begin the infection cycle anew. Five different species of *Plasmodium* cause the majority of human malaria: *P. falciparum*, *P. knowlesi*, *P. malariae*, *P. ovale*, and *P. vivax*. Malaria still accounts for an estimated 445,000 deaths and 216 million cases annually, and the majority of deaths result from infection with *P. falciparum* ^7^.

Malaria deaths have declined in large part because of the development of effective antimalarial medicines. Beginning in the late 1940s, chloroquine was the standard treatment for uncomplicated malaria ^8^. However, by the late 1950s and early 1960s, chloroquine-resistant *P. falciparum* was observed throughout Southeast Asia, Oceania, and South America. Resistance to chloroquine has since spread to nearly all areas of the world ^9^. Subsequent resistance has developed to antimalarials such as sulfadoxine/pyrimethamine, mefloquine, halofantrine, and quinine ^10^. Most recently, resistance to artemisinin-based combination therapies emerged in 2008 in parts of Southeast Asia and continues to spread ^11– 19^. Clinical artemisinin resistance manifests as a delayed clearance phenotype; that is, infection eventually resolves with treatment with artemisinin-based combination therapy, but the time required for parasite clearance substantially increases ^14, 20, 21^. This delayed clearance could contribute to the even more troubling rise in multi-drug resistance, as parasites have gained both reduced artemisinin sensitivity ^22^ and resistance against partner drugs, such as piperaquine ^12, 19^. As multi-drug resistance spreads, there is an urgent need for new antimalarial agents to control malaria infections. Optimally, new antimalarials will overcome multi-drug resistance, will be highly safe for use in vulnerable populations (such as infants and pregnant women), and will target more than one life-cycle stage in order to break the cycle of transmission. In this brief review, we highlight promising novel antimalarials currently in development and introduce emerging drug targets that may be key to ongoing efforts to eliminate malaria worldwide.

## Promising new antimalarials in development

Global efforts to end malaria have led to the development of promising compounds. At the forefront of antimalarial development is the Medicines for Malaria Venture (MMV), which was established in 1999 as a not-for-profit, public–private partnership. The current MMV portfolio contains many promising compounds at various stages of development (research, translational, product development, and access; https://www.mmv.org/research-development/mmv-supported-projects). To illustrate the diversity of the current portfolio, a selection of the emerging antimalarials currently in development is discussed below ( Table 1) ^23– 29^.

**Table 1.** Selected promising antimalarial compounds.

Antimalarial compound	Alternative names	Protein target/predicted target	Target candidate profiles ^a^	Current status in MMV pipeline ^b^
Artefenomel	OZ439	Unknown	Asexual parasite clearance; transmission blocking	Combined artefenomel–ferroquine is in the patient-exploratory stage
Cipargamin	KAE609, NITD609	*Pf*ATP4, based on resistance screen mutations ^23^	Asexual parasite clearance; transmission blocking	Patient-exploratory stage
DSM265	Not applicable	*Plasmodium* DHODH ^24^	Asexual parasite clearance; targeting liver schizonts	Patient-exploratory stage
Ferroquine	SSR97193	Unknown	Asexual parasite clearance; transmission blocking	Combined artefenomel–ferroquine is in the patient-exploratory stage
KAF156	GNF156	*Pf*CARL ^25^, *Pf*ACT, and *Pf*UGT ^26^, based on resistance screen mutations	Asexual parasite clearance; transmission blocking; targeting liver schizonts	Combined KAF156–lumefantrine is in the patient-exploratory stage
MMV048	MMV390048	*Plasmodium* PI4K ^27^	Asexual parasite clearance; transmission blocking; targeting liver schizonts	Patient-exploratory stage
SJ733	(+)-SJ000557733	*Pf*ATP4, based on resistance screen mutations ^28, 29^	Asexual parasite clearance; transmission blocking	Human volunteer stage
Tafenoquine	WR 238605, Etaquine	Unknown	Targeting *Plasmodium* hypnozoites	Regulatory review stage

DHODH, dihydroorotate dehydrogenase PI4K, phosphatidylinositol 4-kinase

^a^According to Medicines for Malaria Venture (MMV) Target Candidate Profile classification.

^b^Status as of 31 July 2018.

### Artefenomel and ferroquine

The current standard of care for malaria is combination therapy based on artemisinin, which is highly valued as a potent, rapidly active antiparasitic compound. Like artemisinin, synthetic ozonides contain an endoperoxide bond. A first-generation ozonide, arterolane (OZ277), has already been licensed for clinical use in India as a combination therapy with piperaquine. However, concern has arisen that there may be a loss of potency against kelch13 mutant parasites, which are artemisinin resistant ^30– 33^. Other synthetic ozonides, including artefenomel (OZ439), have been developed ^34^. Artefenomel displays activity in transmission-blocking assays *in vitro* ^35^, and clinical studies support its use in a single-exposure combination therapy ^36^. Unlike artemisinin’s peroxide bond, artefenomel’s peroxide bond is more stable and has an improved half-life in plasma: 23 hours compared with 0.5 hours ^34^. Promisingly, artemisinin-resistant mutants do not appear to be cross-resistant to artefenomel ^30, 31^, although some mutations in kelch13 may lead to partial cross-resistance ^30– 33^. Together, these properties support the continued efforts toward development and licensure of artefenomel for clinical use.

Ferroquine is a third-generation 4-aminoquinoline and a derivative of the antimalarial chloroquine. Although chloroquine resistance has spread to nearly all areas of the world, ferroquine efficacy is not impeded by chloroquine resistance mechanisms and resistance selection has not been observed in the laboratory ^37^. Ferroquine also retains activity against parasites resistant to chloroquine, mefloquine, quinine, and piperaquine ^38, 39^. An initial clinical study with ferroquine in combination with artesunate showed a high malaria cure rate, and treatment with ferroquine also displayed post-treatment prophylaxis activity for at least 2 months ^40^. Recent phase 2 trials replaced artesunate with the more effective artefenomel ^40– 42^, and a combination of artefenomel and ferroquine therapy is in the patient-exploratory stage of the MMV portfolio.

### DSM265

*Plasmodium* spp. depend on *de novo* pyrimidine synthesis because they lack pyrimidine salvage enzymes. An essential enzyme in the pyrimidine biosynthesis pathway is dihydroorotate dehydrogenase (DHODH). Large high-throughput screens were conducted to identify *Pf*DHODH inhibitors ^43, 44^. These screens identified several classes of molecules that target DHODH, such as triazolopyrimidines, phenylbenzamides, ureas, and naphthamides ^44^. One triazolopyrimidine that has potent activity against asexual and liver-stage parasites is DSM265 ^45^. DSM265 selectively inhibits *Plasmodium* spp. DHODH enzymes over human orthologues ^24^. DSM265 is currently in the patient-exploratory stage of the MMV pipeline and exhibits promising single-dose efficacy. Interestingly, DSM265 is predicted to remain at therapeutic concentrations in humans for more than a week after a single dose because of its favorable pharmacokinetic properties ^45^. DSM265 also has promising prophylactic activity, providing some protection against infection with a single dose up to 7 days before parasite challenge ^46, 47^. The single-dose efficacy of DSM265 makes it exceptionally promising for both prophylaxis and treatment of disease. Furthermore, the potency of DSM265 supports future development of compounds that target *Plasmodium* DHODH.

### Cipargamin and SJ733

Cipargamin (KAE609), a spiroindolone, also has potent activity against blood-stage malaria parasites ^23, 48^. A recent phase 2 study used once-daily dosing of cipargamin for 3 days on 21 adults with either uncomplicated *P. vivax* or *P. falciparum* malaria ^49^. Parasite clearance rates in this clinical study and *in vitro* are among the fastest of any antimalarial yet characterized ^23, 49^. Cipargamin likely targets the P-type Na ^+^ ATPase, *Pf*ATP4, because resistance mutations have emerged ^23^. *Pf*ATP4 appears to regulate parasite ion homeostasis, which is essential for survival, through active Na ^+^ export ^50^. Inhibition of *Pf*ATP4, through cipargamin treatment, perturbs ion homeostasis in the parasite and increases host cell membrane rigidity, resulting in blocked blood-stage development and transmission to mosquitoes ^28, 29, 50– 52^. Cipargamin is currently in the patient-exploratory stage of the MMV portfolio; however, it is not the only *Pf*ATP4 inhibitor currently in development. A diverse range of compounds, including spiroindolones ^23, 51, 53^, pyrazoleamides ^29^, aminopyrazoles ^53^, dihydroisoquinolones ^28^, and other compounds ^54^, have been shown to target *Pf*ATP4. A dihydroisoquinolone that likely targets *Pf*ATP4, SJ733, is in the human volunteer stage of the MMV portfolio. Resistance selection with SJ733, like cipargamin, has generated point mutations, some unique to SJ733 and not induced by cipargamin, in the *pfatp4* gene ^28, 29^. The antimalarial properties of *Pf*ATP4 inhibitors, such as cipargamin and SJ733, are exceptionally promising and support future development of compounds with this mechanism of action.

### KAF156

A novel class of antimalarials, imidazolopiperazines, has recently emerged and been found to have potent asexual blood-stage and liver-stage activity ^55, 56^. One such imidazolopiperazine, KAF156, is currently in the patient-exploratory stage of the MMV portfolio in combination with lumefantrine. Lumefantrine is a clinically approved partner agent; however, it has been modified to a new once-daily formulation for use with KAF156. In addition to displaying asexual blood- and liver-stage activity, KAF156 also inhibits the growth of sexual blood-stage parasites, including mature gametocytes ^57^. Therefore, KAF156 may be effective in preventing parasite transmission from humans to mosquitoes. The antimalarial mechanism of KAF156 is still unclear because resistance *in vitro* is thought to be indirectly mediated through mutation of *pfcarl*, *pfact*, and *pfugt*, which encode a conserved protein of unknown function, an acetyl-CoA transporter, and a UDP-galactose transporter, respectively ^25, 26, 55^. Future studies may illuminate the parasiticidal mechanism of imidazolopiperazines, but this class of drugs has great potential in the treatment of acute disease and reduction of parasite transmission.

### MMV048

Another novel chemical class of antimalarials, 2-aminopyridines, was identified to have potent single-dose activity against *in vitro P. falciparum* and *in vivo P. berghei* ^58^. From this initial screen of 2-aminopyridines, compound 15 (now known as MMV048) was identified with robust antimalarial activity. A follow-up study with MMV048 replicated the potent *in vitro* and *in vivo* activity of asexual blood-stage malaria parasites and also confirmed transmission-blocking and liver-stage activity ^27^. Genomic and chemoproteomic approaches identified *Plasmodium* phosphatidylinositol 4-kinase (PI4K) as the likely target of MMV048 ^27^. PI4K functions in membrane trafficking and membrane assembly during asexual blood-stages ^59^. *Pf*PI4K is likely essential during the asexual blood-stage because attempts to insert an early stop codon were unsuccessful ^59^. The multi-stage antimalarial activity, prolonged half-life, and single-dose efficacy of MMV048 make it a promising new antimalarial in the patient-exploratory stage of the MMV pipeline.

### Tafenoquine

Although infection with *P. falciparum* represents the largest burden of malaria deaths, there is also a need to develop medicines that prevent the relapse of *P. vivax* and *P. ovale*. Unlike *P. falciparum*, both *P. vivax* and *P. ovale* have a dormant liver-stage form called a hypnozoite. Hypnozoites can reactivate without warning, leading to the onset of malarial symptoms. This dormant stage remains both a challenge to treat and a potent barrier to malaria elimination. Tafenoquine, an 8-aminoquinoline, is currently under development for the prevention of *P. vivax* relapse. Tafenoquine has high activity as a single-dose treatment and has promising anti-hypnozoite activity in humans ^60^. However, tafenoquine has therapeutic restrictions similar to those of the current radical cure standard for *P. vivax* and *P. ovale*, primaquine, which might limit its therapeutic impact. Both primaquine and tafenoquine cause dose-dependent acute hemolytic anemia in individuals with glucose-6-phosphate dehydrogenase deficiency ^61, 62^. Because of its longer half-life, tafenoquine requires a higher glucose-6-phosphate dehydrogenase activity threshold than primaquine; thus, a greater proportion of individuals will be ineligible for tafenoquine treatment and primaquine will still be needed ^63^. In July 2018, the US Food and Drug Administration approved tafenoquine under the trade name Krintafel. Tafenoquine is the first new antimalarial in 60 years to prevent relapse of *P. vivax.* However, its limitations highlight the need for development of additional compounds that target relapsing malaria.

## Emerging new antimalarial targets

Continued efforts to dissect the basic biology of the complex malarial organism have yielded new therapeutic targets for the development of antimalarials. The *Plasmodium* Genetic Modification Project ( *Plasmo*GEM) ( http://plasmogem.sanger.ac.uk), a not-for-profit, open-access research resource, has advanced our understanding of *Plasmodium* by providing vectors for genome-wide manipulation. *Plasmo*GEM contains over 2,000 plasmids designed to tag or delete genes in *P. berghei* ^64^. Provided without cost, these tools have been used in a recent large-scale knockout screen to identify essential genes. The essentiality of over 50% of the genome was tested in an *in vivo* mouse model of *P. berghei* infection ^65^. Surprisingly, 44.9% of genes were found to be essential and an additional 18% showed reduced parasite blood-stage growth; therefore, 62.9% of genes are required in *P. berghei* for normal asexual growth ^65^. The high percentage of essential genes and low functional redundancy suggest that *Plasmodium* may have considerably more drug targets than do bacteria, for example ^65^. This genetic screen resulted in the identification of essential cellular processes in the parasite. Specific examples of pathways enriched with essential genes include glycosylphosphatidylinositol anchor biosynthesis, the mitochondrial tricarboxylic acid cycle, ubiquinone biosynthesis, and isoprenoid biosynthesis ^65^. From this genetic screen, a searchable phenotype database was built ( http://plasmogem.sanger.ac.uk/phenotypes).

A large forward genetic screen in *P. falciparum* parasites recently identified more than 2,680 genes that are likely essential for asexual blood-stage growth ^66^. When high-throughput *piggyBac* transposon insertional mutagenesis was used in combination with quantitative insertion site sequencing ^67, 68^, the mutability and fitness cost of 5,399 genes were evaluated ^66^. The AT-richness of the *P. falciparum* genome (>81%) is well suited for *piggyBac* transposon-based mutagenesis because of the high density of the tetranucleotide insertion target sequence TTAA ^66^. To quantify gene essentiality, a mutagenesis index score and mutagenesis fitness score were calculated for each locus. Together, these two independent measures were used to classify a gene as likely essential or dispensable, and this methodology may be expanded to identify essential genes for other life-cycle stages ^66^. The essential genes and pathways discovered in both the *Plasmo*GEM *P. berghei* screen ^65^ and *piggyBac* transposon *P. falciparum* screen ^66^ will supplement ongoing studies and likely initiate investigation into novel putative antimalarial targets.

**Figure 1.**
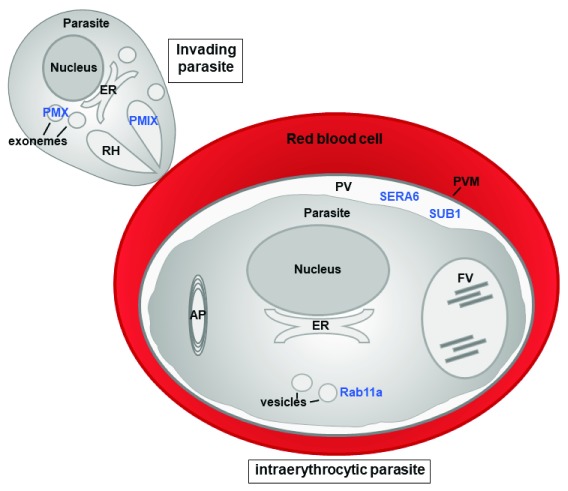
Localization of antimalarial targets in the asexual parasite. Shown are parasite organelles, including the nucleus, apicoplast (AP), endoplasmic reticulum (ER), food vacuole (FV), rhoptries (RH), exonemes, and vesicles. The intraerythrocytic parasite is located within the parasitophorous vacuole (PV), which is delineated by the PV membrane (PVM), where both SUB1 and SERA6 are found during egress ^98^. Rab11a likely localizes to vesicles, which it guides to target membranes. In the invading parasite, plasmepsin IX (PMIX) is found in the bulbs of RH and plasmepsin X (PMX) localizes to exonemes ^72^.

A final strategy to identify possible new antimalarial targets is the resistance screen. Resistance screens challenge the malaria parasite with low levels of an antimalarial compound to hinder development. This can lead to *in vitro* evolution and selection for resistance mutations that relieve growth suppression. Therefore, resistance screens can be used to discover both mediators of drug resistance and novel antimalarial drug targets ^22, 69^. Winzeler and colleagues recently performed a large resistance screen with 37 distinct compounds ^70^. Whole genome sequencing of 262 compound-resistant parasite lines identified several candidate resistance mutations. Although the screen confirmed previously identified multi-drug resistance mechanisms and illuminated new drug target–inhibitor pairs, only two novel drug-resistance genes— *pfabcI3* and *pfaat1* ^70^—were identified. Below, we highlight a few emerging antimalarial targets of particular promise ( Figure 1).

### PMIX and PMX

Two recent studies highlighted the importance of plasmepsins IX (PMIX) and X (PMX) for parasite development ^71, 72^. Plasmepsins comprise a family of 10 aspartic proteases in the *P. falciparum* genome. A number of studies have focused on the digestive vacuole plasmepsins I to IV (PMI to PMIV), including development of chemical inhibitors ^73– 76^. Subsequent functional genetic studies of PMI–PMIV revealed that they are not essential for parasite survival ^77^. PMIX and PMX are expressed in asexual blood-stage parasites ^78^. Conditional knockdown of PMIX in *P. falciparum* revealed that it is essential for red blood cell invasion ^71, 72^. PMX knockdown similarly interrupted red blood cell invasion but also revealed an additional requirement of PMX in red blood cell egress ^72^. A recent study employed a combination of *in vitro* and *in vivo* rodent experiments to find that hydroxyl-ethyl-amine-based scaffold compound 49c (referred to as 49c) inhibits both PMIX and PMX ^71^. 49c is an effective inhibitor against *P. falciparum in vitro* and the rodent parasite *Plasmodium berghei in vivo* ^79, 80^. Treatment with 49c inhibits asexual, sexual, and liver-stage development, indicating that 49c or other PMIX or PMX inhibitors may have value to both treat symptomatic malaria and block transmission ^71^. Together, these observations provide compelling evidence that PMIX and PMX are promising targets for antimalarial development.

### Rab11a

In the recent *P. berghei* functional genetic screen, one of the cellular pathways most enriched with essential genes is that of isoprenoid biosynthesis ^65^. A number of studies have previously highlighted the requirement of isoprenoid biosynthesis for *P. falciparum* asexual replication ^81– 83^. Isoprenoids are necessary for protein prenylation, the post-translational lipid modification of proteins. Because chemical inhibition of protein prenylation in the malaria parasite disrupts asexual parasite growth ^84– 90^, prenylated malarial proteins are potential antimalarial targets. Recent chemical labeling approaches have revealed that only 15 to 19 proteins are prenylated in blood-stage malaria and a majority of these proteins are Rab GTPases ^91, 92^. Rab GTPases function in docking vesicles to membranes and their prenylation aids in association with target membranes ^93^. Rab11a, a Rab GTPase, is expressed and prenylated in asexual blood-stage malaria parasites ^91, 92, 94^ and is essential for asexual parasite replication ^95^. In the parasite, Rab11a functions as a mediator of PI4K signaling and is a binding partner of PI4K, the target of imidazopyrazines and MMV048 ^27, 59, 96^. Mutation of Rab11a confers resistance to the imidazopyrazine, KAI715 ^59^. Interestingly, Rab11a has very low genetic diversity when sequenced in 2,000 *Plasmodium* clinical isolates, and only one non-synonymous mutation has been identified ^97^. These features suggest that Rab11a may represent a promising target because of both its prenylation and interactions with essential signaling pathways within the parasite. Additional studies are needed to evaluate the biological roles of the remaining prenylated proteins in blood-stage malaria.

### SUB1 and SERA6

During asexual replication, the malaria parasite must exit the red blood cell before invading a new cell. This process, called egress, requires the rupture of both the parasitophorous vacuole membrane (PVM), which surrounds the parasite, and the red blood cell membrane (RBCM). Egress is protease dependent ^99^, and recently two proteases—SUB1 and SERA6—were identified as mediators of PVM and RBCM rupture ^98^. SUB1, a serine protease, moves to the parasitophorous vacuole before egress ^100– 104^ and cleaves multiple substrates ^100, 103, 105– 107^. One substrate cleaved by SUB1 is SERA6 ^108– 110^, a putative cysteine protease, which requires proteolytic processing by SUB1 to function ^98^. *P. falciparum* parasites that lack SUB1 fail to rupture the PVM, thus stalling parasite development ^98^. Interestingly, parasites that lack SERA6 can rupture the PVM, but RBCM rupture does not occur ^98^. Therefore, SUB1 and SERA6 have distinct roles in parasite egress. Because SUB1 and SERA6 are essential for asexual blood-stage growth and orthologues are found in other *Plasmodium* species ^98^, compounds that inhibit these proteins may be useful in treating multiple types of malarial disease.

## Discussion

Malaria continues to be a major global health concern. *Plasmodium* elimination will not be possible without substantial ongoing efforts, including diagnostic testing and treatment of confirmed and asymptomatic infections, mosquito vector control, preventative therapies, and surveillance systems. Although all of these areas of malaria control are crucial, antimalarial drug discovery is among the most pressing because of the continued spread of antimalarial resistance. The MMV has focused its efforts on developing drugs that treat disease, prevent transmission, and provide chemoprotection. This multifaceted approach to the antimalarial development pipeline provides assurance that new antimalarials will contribute to broad approaches of malaria control.

Studies in basic parasite biology remain extremely important to elimination efforts. Although the current antimalarial compounds under development have great potential, malaria control efforts will benefit from continued dissection of parasite biology. Ideally, new compounds will target proteins and pathways that are essential for parasite growth and transmission with diverse mechanisms of action. New antimalarials will almost certainly be employed in combination therapeutics, combining molecules of different chemical classes and with diverse mechanisms of action to slow the development of multi-drug resistance. Novel drug targets will be uncovered through multiple approaches, such as large-scale genetic and resistance screens. A deeper understanding of essential parasite biology will also aid in other aspects of malaria control, including mosquito vector control, advancement of diagnostic tests, and development of preventative therapies. To date, *Plasmodium* has successfully adapted to sequential drug selective pressure in the field. However, the remarkable successes of recent efforts to develop new antimalarials and identify drug targets suggest an optimistic future in treatment of disease, prevention of transmission, and protection against infection.

## Abbreviations

49c, hydroxyl-ethyl-amine-based scaffold compound 49c; DHODH, dihydroorotate dehydrogenase; MMV, Medicines for Malaria Venture; PI4K, phosphatidylinositol 4-kinase; *Plasmo*GEM, *Plasmodium* Genetic Modification Project; PMI, plasmepsin I; PMIV, plasmepsin IV; PMIX, plasmepsin IX; PMX, plasmepsin X; PVM, parasitophorous vacuole membrane; RBCM, red blood cell membrane

## References

[1] Laveran A: Un nouveau parasite trouvé dans le sang de malades atteints de fièvre palustre. Origine parasitaire des accidents de l’impaludisme. *Bull Mém Soc Méd Hôpitaux Paris.* 1881;17:158–164. Reference Source

[2] Manson P: SURGEON-MAJOR RONALD ROSS'S RECENT INVESTIGATIONS on the MOSQUITO-MALARIA THEORY. *Br Med J.* 1898;1(1995):1575–7. 10.1136/bmj.1.1955.1575 20757898 PMC2411754

[3] Ross R: The role of the mosquito in the evolution of the malarial parasite: the recent researches of Surgeon-Major Ronald Ross, I.M.S. 1898. *Yale J Biol Med.* 2002;75(2):103–5. 12230308 PMC2588727

[4] Golgi C: Sul’ infezione malarica. *Arch Sci Med Torino.* 1886;10:109–135.

[5] Golgi C: Sul ciclo evolutivo dei parassiti malarici nella febbre terzana: diagnosi differenziale tra i parassiti endoglobulari malarici della terzana e quelli della quartana. *Arch Sci Med Torino.* 1889;13:173–196.

[6] Grassi B Bignami A Bastianelli G: Ulteriore ricerche sul ciclo dei parassiti malarici umani sul corpo del zanzarone. *Atti R Accad Lincei.* 1899;8(21–28).

[7] World Health Organization: World Malaria Report 2017.Geneva; licence: CC BY-NC-SA 3.0 IGO.2017 Reference Source

[8] Coatney GR: Pitfalls in a Discovery: The Chronicle of Chloroquine. *Am J Trop Med Hyg.* 1963;12:121–8. 10.4269/ajtmh.1963.12.121 14021822

[9] Ridley RG: Medical need, scientific opportunity and the drive for antimalarial drugs. *Nature.* 2002;415(6872):686–93. 10.1038/415686a 11832957

[10] Baird JK: Effectiveness of antimalarial drugs. *N Engl J Med.* 2005;352(15):1565–77. 10.1056/NEJMra043207 15829537

[11] Huang F Takala-Harrison S Jacob CG: A Single Mutation in K13 Predominates in Southern China and Is Associated With Delayed Clearance of *Plasmodium falciparum* Following Artemisinin Treatment. *J Infect Dis.* 2015;212(10):1629–35. 10.1093/infdis/jiv249 25910630 PMC4621243

[12] Amaratunga C Sreng S Suon S: Artemisinin-resistant *Plasmodium falciparum* in Pursat province, western Cambodia: a parasite clearance rate study. *Lancet Infect Dis.* 2012;12(11):851–8. 10.1016/S1473-3099(12)70181-0 22940027 PMC3786328

[13] Phyo AP Nkhoma S Stepniewska K: Emergence of artemisinin-resistant malaria on the western border of Thailand: a longitudinal study. *Lancet.* 2012;379(9830):1960–6. 10.1016/S0140-6736(12)60484-X 22484134 PMC3525980

[14] Dondorp AM Nosten F Yi P: Artemisinin resistance in *Plasmodium falciparum* malaria. *N Engl J Med.* 2009;361(5):455–67. 10.1056/NEJMoa0808859 19641202 PMC3495232

[15] Thriemer K Hong VN Rosanas-Urgell A: Delayed parasite clearance after treatment with dihydroartemisinin-piperaquine in *Plasmodium falciparum* malaria patients in central Vietnam. *Antimicrob Agents Chemother.* 2014;58(12):7049–55. 10.1128/AAC.02746-14 25224002 PMC4249535

[16] MalariaGEN Plasmodium falciparum Community Project: Genomic epidemiology of artemisinin resistant malaria. *eLife.* 2016; pii: e08714. 10.7554/eLife.08714 26943619 PMC4786412

[17] Kyaw MP Nyunt MH Chit K: Reduced susceptibility of *Plasmodium falciparum* to artesunate in southern Myanmar. *PLoS One.* 2013;8(3):e57689. 10.1371/journal.pone.0057689 23520478 PMC3592920

[18] Hien TT Thuy-Nhien NT Phu NH: *In vivo* susceptibility of *Plasmodium falciparum* to artesunate in Binh Phuoc Province, Vietnam. *Malar J.* 2012;11:355. 10.1186/1475-2875-11-355 23101492 PMC3504531

[19] Leang R Taylor WR Bouth DM: Evidence of *Plasmodium falciparum* Malaria Multidrug Resistance to Artemisinin and Piperaquine in Western Cambodia: Dihydroartemisinin-Piperaquine Open-Label Multicenter Clinical Assessment. *Antimicrob Agents Chemother.* 2015;59(8):4719–26. 10.1128/AAC.00835-15 26014949 PMC4505193

[20] Dondorp AM Fairhurst RM Slutsker L: The threat of artemisinin-resistant malaria. *N Engl J Med.* 2011;365(12):1073–5. 10.1056/NEJMp1108322 21992120 PMC3733336

[21] Noedl H Se Y Schaecher K: Evidence of artemisinin-resistant malaria in western Cambodia. *N Engl J Med.* 2008;359(24):2619–20. 10.1056/NEJMc0805011 19064625

[22] Ariey F Witkowski B Amaratunga C: A molecular marker of artemisinin-resistant *Plasmodium falciparum* malaria. *Nature.* 2014;505(7481):50–5. 10.1038/nature12876 24352242 PMC5007947

[23] Rottmann M McNamara C Yeung BK: Spiroindolones, a potent compound class for the treatment of malaria. *Science.* 2010;329(5996):1175–80. 10.1126/science.1193225 20813948 PMC3050001

[24] Coteron JM Marco M Esquivias J: Structure-guided lead optimization of triazolopyrimidine-ring substituents identifies potent *Plasmodium falciparum* dihydroorotate dehydrogenase inhibitors with clinical candidate potential. *J Med Chem.* 2011;54(15):5540–61. 10.1021/jm200592f 21696174 PMC3156099

[25] LaMonte G Lim MY Wree M: Mutations in the *Plasmodium falciparum* Cyclic Amine Resistance Locus (PfCARL) Confer Multidrug Resistance. *mBio.* 2016;7(4): pii: e00696-16. 10.1128/mBio.00696-16 27381290 PMC4958248

[26] Lim MY LaMonte G Lee MC: UDP-galactose and acetyl-CoA transporters as *Plasmodium* multidrug resistance genes. *Nat Microbiol.* 2016;1; 16166. 10.1038/nmicrobiol.2016.166 27642791 PMC5575994

[27] Paquet T Le Manach C Cabrera DG: Antimalarial efficacy of MMV390048, an inhibitor of *Plasmodium* phosphatidylinositol 4-kinase. *Sci Transl Med.* 2017;9(387): pii: eaad9735. 10.1126/scitranslmed.aad9735 28446690 PMC5731459

[28] Jiménez-Díaz MB Ebert D Salinas Y: (+)-SJ733, a clinical candidate for malaria that acts through ATP4 to induce rapid host-mediated clearance of *Plasmodium*. *Proc Natl Acad Sci U S A.* 2014;111(50):E5455-62. 10.1073/pnas.1414221111 25453091 PMC4273362

[29] Vaidya AB Morrisey JM Zhang Z: Pyrazoleamide compounds are potent antimalarials that target Na ^+^ homeostasis in intraerythrocytic *Plasmodium falciparum*. *Nat Commun.* 2014;5: 5521. 10.1038/ncomms6521 25422853 PMC4263321

[30] Straimer J Gnädig NF Stokes BH: *Plasmodium falciparum* K13 Mutations Differentially Impact Ozonide Susceptibility and Parasite Fitness *In Vitro*. *mBio.* 2017;8(2): pii: e00172-17. 10.1128/mBio.00172-17 28400526 PMC5388803

[31] Siriwardana A Iyengar K Roepe PD: Endoperoxide Drug Cross-Resistance Patterns for *Plasmodium falciparum* Exhibiting an Artemisinin Delayed-Clearance Phenotype. *Antimicrob Agents Chemother.* 2016;60(11):6952–6. 10.1128/AAC.00857-16 27600038 PMC5075116

[32] Yang T Xie SC Cao P: Comparison of the Exposure Time Dependence of the Activities of Synthetic Ozonide Antimalarials and Dihydroartemisinin against K13 Wild-Type and Mutant *Plasmodium falciparum* Strains. *Antimicrob Agents Chemother.* 2016;60(8):4501–10. 10.1128/AAC.00574-16 27161632 PMC4958167

[33] Baumgärtner F Jourdan J Scheurer C: *In vitro* activity of anti-malarial ozonides against an artemisinin-resistant isolate. *Malar J.* 2017;16(1):45. 10.1186/s12936-017-1696-0 28122617 PMC5267415

[34] Charman SA Arbe-Barnes S Bathurst IC: Synthetic ozonide drug candidate OZ439 offers new hope for a single-dose cure of uncomplicated malaria. *Proc Natl Acad Sci U S A.* 2011;108(11):4400–5. 10.1073/pnas.1015762108 21300861 PMC3060245

[35] Bolscher JM Koolen KM van Gemert GJ: A combination of new screening assays for prioritization of transmission-blocking antimalarials reveals distinct dynamics of marketed and experimental drugs. *J Antimicrob Chemother.* 2015;70(5):1357–66. 10.1093/jac/dkv003 25667405

[36] McCarthy JS Baker M O'Rourke P: Efficacy of OZ439 (artefenomel) against early *Plasmodium falciparum* blood-stage malaria infection in healthy volunteers. *J Antimicrob Chemother.* 2016;71(9):2620–7. 10.1093/jac/dkw174 27272721 PMC4992851

[37] Daher W Biot C Fandeur T: Assessment of *Plasmodium falciparum* resistance to ferroquine (SSR97193) in field isolates and in W2 strain under pressure. *Malar J.* 2006;5:11. 10.1186/1475-2875-5-11 16464254 PMC1395321

[38] Henry M Briolant S Fontaine A: *In vitro* activity of ferroquine is independent of polymorphisms in transport protein genes implicated in quinoline resistance in *Plasmodium falciparum*. *Antimicrob Agents Chemother.* 2008;52(8):2755–9. 10.1128/AAC.00060-08 18505855 PMC2493139

[39] Barends M Jaidee A Khaohirun N: *In vitro* activity of ferroquine (SSR 97193) against *Plasmodium falciparum* isolates from the Thai-Burmese border. *Malar J.* 2007;6:81. 10.1186/1475-2875-6-81 17597537 PMC1934364

[40] Held J Supan C Salazar CL: Ferroquine and artesunate in African adults and children with *Plasmodium falciparum* malaria: A phase 2, multicentre, randomised, double-blind, dose-ranging, non-inferiority study. *Lancet Infect Dis.* 2015;15(12):1409–19. 10.1016/S1473-3099(15)00079-1 26342427

[41] Wells TN Hooft van Huijsduijnen R: Ferroquine: Welcome to the next generation of antimalarials. *Lancet Infect Dis.* 2015;15:1365–6. 10.1016/S1473-3099(15)00148-6 26342426

[42] McCarthy JS Rückle T Djeriou E: A Phase II pilot trial to evaluate safety and efficacy of ferroquine against early *Plasmodium falciparum* in an induced blood-stage malaria infection study. *Malar J.* 2016;15(1):469. 10.1186/s12936-016-1511-3 27624471 PMC5022189

[43] Patel V Booker M Kramer M: Identification and characterization of small molecule inhibitors of *Plasmodium falciparum* dihydroorotate dehydrogenase. *J Biol Chem.* 2008;283:35078–85. 10.1074/jbc.M804990200 18842591 PMC2596402

[44] Baldwin J Michnoff CH Malmquist NA: High-throughput screening for potent and selective inhibitors of *Plasmodium falciparum* dihydroorotate dehydrogenase. *J Biol Chem.* 2005;280(23):21847–53. 10.1074/jbc.M501100200 15795226

[45] Phillips MA Lotharius J Marsh K: A long-duration dihydroorotate dehydrogenase inhibitor (DSM265) for prevention and treatment of malaria. *Sci Transl Med.* 2015;7(296):296ra111. 10.1126/scitranslmed.aaa6645 26180101 PMC4539048

[46] Sulyok M Rückle T Roth A: DSM265 for *Plasmodium falciparum* chemoprophylaxis: A randomised, double blinded, phase 1 trial with controlled human malaria infection. *Lancet Infect Dis.* 2017;17(6):636–44. 10.1016/S1473-3099(17)30139-1 28363637 PMC5446410

[47] Murphy SC Duke ER Shipman KJ: A Randomized Trial Evaluating the Prophylactic Activity of DSM265 Against Preerythrocytic *Plasmodium falciparum* Infection During Controlled Human Malarial Infection by Mosquito Bites and Direct Venous Inoculation. *J Infect Dis.* 2018;217(5):693–702. 10.1093/infdis/jix613 29216395 PMC5853383

[48] Yeung BK Zou B Rottmann M: Spirotetrahydro beta-carbolines (spiroindolones): a new class of potent and orally efficacious compounds for the treatment of malaria. *J Med Chem.* 2010;53(14):5155–64. 10.1021/jm100410f 20568778 PMC6996867

[49] White NJ Pukrittayakamee S Phyo AP: Spiroindolone KAE609 for falciparum and vivax malaria. *N Engl J Med.* 2014;371(5):403–10. 10.1056/NEJMoa1315860 25075833 PMC4143746

[50] Kirk K: Ion Regulation in the Malaria Parasite. *Annu Rev Microbiol.* 2015;69:341–59. 10.1146/annurev-micro-091014-104506 26488277

[51] Spillman NJ Allen RJ McNamara CW: Na ^+^ regulation in the malaria parasite *Plasmodium falciparum* involves the cation ATPase PfATP4 and is a target of the spiroindolone antimalarials. *Cell Host Microbe.* 2013;13(2):227–37. 10.1016/j.chom.2012.12.006 23414762 PMC3574224

[52] van Pelt-Koops JC Pett HE Graumans W: The spiroindolone drug candidate NITD609 potently inhibits gametocytogenesis and blocks *Plasmodium falciparum* transmission to anopheles mosquito vector. *Antimicrob Agents Chemother.* 2012;56(7):3544–8. 10.1128/AAC.06377-11 22508309 PMC3393464

[53] Flannery EL McNamara CW Kim SW: Mutations in the P-type cation-transporter ATPase 4, PfATP4, mediate resistance to both aminopyrazole and spiroindolone antimalarials. *ACS Chem Biol.* 2015;10(2):413–20. 10.1021/cb500616x 25322084 PMC4340351

[54] Lehane AM Ridgway MC Baker E: Diverse chemotypes disrupt ion homeostasis in the Malaria parasite. *Mol Microbiol.* 2014;94(2):327–39. 10.1111/mmi.12765 25145582

[55] Meister S Plouffe DM Kuhen KL: Imaging of *Plasmodium* liver stages to drive next-generation antimalarial drug discovery. *Science.* 2011;334(6061):1372–7. 10.1126/science.1211936 22096101 PMC3473092

[56] Wu T Nagle A Kuhen K: Imidazolopiperazines: hit to lead optimization of new antimalarial agents. *J Med Chem.* 2011;54(14):5116–30. 10.1021/jm2003359 21644570 PMC6950218

[57] Kuhen KL Chatterjee AK Rottmann M: KAF156 is an antimalarial clinical candidate with potential for use in prophylaxis, treatment, and prevention of disease transmission. *Antimicrob Agents Chemother.* 2014;58(9):5060–7. 10.1128/AAC.02727-13 24913172 PMC4135840

[58] Younis Y Douelle F Feng TS: 3,5-Diaryl-2-aminopyridines as a novel class of orally active antimalarials demonstrating single dose cure in mice and clinical candidate potential. *J Med Chem.* 2012;55(7):3479–87. 10.1021/jm3001373 22390538

[59] McNamara CW Lee MC Lim CS: Targeting *Plasmodium* PI(4)K to eliminate malaria. *Nature.* 2013;504(7479):248–53. 10.1038/nature12782 24284631 PMC3940870

[60] Beck HP Wampfler R Carter N: Estimation of the Antirelapse Efficacy of Tafenoquine, Using *Plasmodium vivax* Genotyping. *J Infect Dis.* 2016;213(5):794–9. 10.1093/infdis/jiv508 26500351 PMC4747625

[61] Rueangweerayut R Bancone G Harrell EJ: Hemolytic Potential of Tafenoquine in Female Volunteers Heterozygous for Glucose-6-Phosphate Dehydrogenase (G6PD) Deficiency ( *G6PD Mahidol* Variant) versus G6PD-Normal Volunteers. *Am J Trop Med Hyg.* 2017;97(3):702–11. 10.4269/ajtmh.16-0779 28749773 PMC5590573

[62] ALVING AS JOHNSON CF TARLOV AR: Mitigation of the haemolytic effect of primaquine and enhancement of its action against exoerythrocytic forms of the Chesson strain of Piasmodium vivax by intermittent regimens of drug administration: a preliminary report. *Bull World Health Organ.* 1960;22:621–31. 13793053 PMC2555355

[63] Watson J Taylor WRJ Bancone G: Implications of current therapeutic restrictions for primaquine and tafenoquine in the radical cure of vivax malaria. *PLoS Negl Trop Dis.* 2018;12(4):e0006440. 10.1371/journal.pntd.0006440 29677199 PMC5931686

[64] Schwach F Bushell E Gomes AR: *Plasmo*GEM, a database supporting a community resource for large-scale experimental genetics in malaria parasites. *Nucleic Acids Res.* 2015;43(Database issue):D1176–82. 10.1093/nar/gku1143 25593348 PMC4383951

[65] Bushell E Gomes AR Sanderson T: Functional Profiling of a *Plasmodium* Genome Reveals an Abundance of Essential Genes. *Cell.* 2017;170(2):260–272.e8. 10.1016/j.cell.2017.06.030 28708996 PMC5509546

[66] Zhang M Wang C Otto TD: Uncovering the essential genes of the human malaria parasite *Plasmodium falciparum* by saturation mutagenesis. *Science.* 2018;360(6388): pii: eaap7847. 10.1126/science.aap7847 29724925 PMC6360947

[67] Bronner IF Otto TD Zhang M: Quantitative insertion-site sequencing (QIseq) for high throughput phenotyping of transposon mutants. *Genome Res.* 2016;26(7):980–9. 10.1101/gr.200279.115 27197223 PMC4937560

[68] Hart T Chandrashekhar M Aregger M: High-Resolution CRISPR Screens Reveal Fitness Genes and Genotype-Specific Cancer Liabilities. *Cell.* 2015;163(6):1515–26. 10.1016/j.cell.2015.11.015 26627737

[69] Flannery EL Fidock DA Winzeler EA: Using genetic methods to define the targets of compounds with antimalarial activity. *J Med Chem.* 2013;56(20):7761–71. 10.1021/jm400325j 23927658 PMC3880619

[70] Cowell AN Istvan ES Lukens AK: Mapping the malaria parasite druggable genome by using *in vitro* evolution and chemogenomics. *Science.* 2018;359(6372):191–9. 10.1126/science.aan4472 29326268 PMC5925756

[71] Pino P Caldelari R Mukherjee B: A multistage antimalarial targets the plasmepsins IX and X essential for invasion and egress. *Science.* 2017;358(6362):522–8. 10.1126/science.aaf8675 29074775 PMC5730047

[72] Nasamu AS Glushakova S Russo I: Plasmepsins IX and X are essential and druggable mediators of malaria parasite egress and invasion. *Science.* 2017;358(6362):518–22. 10.1126/science.aan1478 29074774 PMC5928414

[73] Bhaumik P Gustchina A Wlodawer A: Structural studies of vacuolar plasmepsins. *Biochim Biophys Acta.* 2012;1824(1):207–23. 10.1016/j.bbapap.2011.04.008 21540129 PMC3154504

[74] Meyers MJ Goldberg DE: Recent advances in plasmepsin medicinal chemistry and implications for future antimalarial drug discovery efforts. *CTMC.* 2012;12(5):445–55. 10.2174/156802612799362959 22242846 PMC11670882

[75] Boss C Richard-Bildstein S Weller T: Inhibitors of the *Plasmodium falciparum* parasite aspartic protease plasmepsin II as potential antimalarial agents. *CMC.* 2003;10(11):883–907. 10.2174/0929867033457674 12678679

[76] Rasina D Otikovs M Leitans J: Fragment-Based Discovery of 2-Aminoquinazolin-4(3 *H*)-ones As Novel Class Nonpeptidomimetic Inhibitors of the Plasmepsins I, II, and IV. *J Med Chem.* 2016;59(1):374–87. 10.1021/acs.jmedchem.5b01558 26670264

[77] Bonilla JA Bonilla TD Yowell CA: Critical roles for the digestive vacuole plasmepsins of *Plasmodium falciparum* in vacuolar function. *Mol Microbiol.* 2007;65(1):64–75. 10.1111/j.1365-2958.2007.05768.x 17581121

[78] Banerjee R Liu J Beatty W: Four plasmepsins are active in the *Plasmodium falciparum* food vacuole, including a protease with an active-site histidine. *Proc Natl Acad Sci U S A.* 2002;99(2):990–5. 10.1073/pnas.022630099 11782538 PMC117418

[79] Ciana C Siegrist R Aissaoui H: Novel *in vivo* active anti-malarials based on a hydroxy-ethyl-amine scaffold. *Bioorg Med Chem Lett.* 2013;23(3):658–62. 10.1016/j.bmcl.2012.11.118 23260352

[80] Guiguemde WA Shelat AA Bouck D: Chemical genetics of *Plasmodium falciparum*. *Nature.* 2010;465(7296):311–5. 10.1038/nature09099 20485428 PMC2874979

[81] Zhang B Watts KM Hodge D: A second target of the antimalarial and antibacterial agent fosmidomycin revealed by cellular metabolic profiling. *Biochemistry.* 2011;50(17):3570–7. 10.1021/bi200113y 21438569 PMC3082593

[82] Yeh E DeRisi JL: Chemical rescue of malaria parasites lacking an apicoplast defines organelle function in blood-stage *Plasmodium falciparum*. *PLoS Biol.* 2011;9(8):e1001138. 10.1371/journal.pbio.1001138 21912516 PMC3166167

[83] Jomaa H Wiesner J Sanderbrand S: Inhibitors of the Nonmevalonate Pathway of Isoprenoid Biosynthesis as Antimalarial Drugs. *Science.* 1999;285(5433):1573–6. 10.1126/science.285.5433.1573 10477522

[84] Buckner FS Eastman RT Yokoyama K: Protein farnesyl transferase inhibitors for the treatment of malaria and African trypanosomiasis. *Curr Opin Investig Drugs.* 2005;6(8):791–7. 16121685

[85] Glenn MP Chang SY Hucke O: Structurally simple farnesyltransferase inhibitors arrest the growth of malaria parasites. *Angew Chem Int Ed Engl.* 2005;44(31):4903–6. 10.1002/anie.200500674 16007716

[86] Glenn MP Chang SY Hornéy C: Structurally simple, potent, *Plasmodium* selective farnesyltransferase inhibitors that arrest the growth of malaria parasites. *J Med Chem.* 2006;49(19):5710–27. 10.1021/jm060081v 16970397 PMC2728208

[87] Nallan L Bauer KD Bendale P: Protein farnesyltransferase inhibitors exhibit potent antimalarial activity. *J Med Chem.* 2005;48(11):3704–13. 10.1021/jm0491039 15916422

[88] Howe R Kelly M Jimah J: Isoprenoid biosynthesis inhibition disrupts Rab5 localization and food vacuolar integrity in *Plasmodium falciparum*. *Eukaryot Cell.* 2013;12(2):215–23. 10.1128/EC.00073-12 23223036 PMC3571303

[89] Chakrabarti D Da Silva T Barger J: Protein farnesyltransferase and protein prenylation in *Plasmodium falciparum*. *J Biol Chem.* 2002;277(44):42066–73. 10.1074/jbc.M202860200 12194969

[90] Chakrabarti D Azam T DelVecchio C: Protein prenyl transferase activities of *Plasmodium falciparum*. *Mol Biochem Parasitol.* 1998;94(2):175–84. 10.1016/S0166-6851(98)00065-6 9747968

[91] Gisselberg JE Zhang L Elias JE: The Prenylated Proteome of *Plasmodium falciparum* Reveals Pathogen-specific Prenylation Activity and Drug Mechanism-of-action. *Mol Cell Proteomics.* 2017;16(4 suppl 1):S54–S64. 10.1074/mcp.M116.064550 28040698 PMC5393391

[92] Suazo KF Schaber C Palsuledesai CC: Global proteomic analysis of prenylated proteins in *Plasmodium falciparum* using an alkyne-modified isoprenoid analogue. *Sci Rep.* 2016;6:38615. 10.1038/srep38615 27924931 PMC5141570

[93] Gomes AQ Ali BR Ramalho JS: Membrane targeting of Rab GTPases is influenced by the prenylation motif. *Mol Biol Cell.* 2003;14(5):1882–99. 10.1091/mbc.e02-10-0639 12802062 PMC165084

[94] Quevillon E Spielmann T Brahimi K: The *Plasmodium falciparum* family of Rab GTPases. *Gene.* 2003;306:13–25. 10.1016/S0378-1119(03)00381-0 12657463

[95] Agop-Nersesian C Naissant B Ben Rached F: Rab11A-controlled assembly of the inner membrane complex is required for completion of apicomplexan cytokinesis. *PLoS Pathog.* 2009;5(1):e1000270. 10.1371/journal.ppat.1000270 19165333 PMC2622761

[96] Burke JE Inglis AJ Perisic O: Structures of PI4KIIIβ complexes show simultaneous recruitment of Rab11 and its effectors. *Science.* 2014;344(6187):1035–8. 10.1126/science.1253397 24876499 PMC4046302

[97] Gomes AR Ravenhall M Benavente ED: Genetic diversity of next generation antimalarial targets: A baseline for drug resistance surveillance programmes. *Int J Parasitol Drugs Drug Resist.* 2017;7(2):174–80. 10.1016/j.ijpddr.2017.03.001 28376349 PMC5379905

[98] Thomas JA Tan MSY Bisson C: A protease cascade regulates release of the human malaria parasite *Plasmodium falciparum* from host red blood cells. *Nat Microbiol.* 2018;3(4):447–55. 10.1038/s41564-018-0111-0 29459732 PMC6089347

[99] Blackman MJ: Malarial proteases and host cell egress: an 'emerging' cascade. *Cell Microbiol.* 2008;10(10):1925–34. 10.1111/j.1462-5822.2008.01176.x 18503638 PMC2610400

[100] Yeoh S O'Donnell RA Koussis K: Subcellular discharge of a serine protease mediates release of invasive malaria parasites from host erythrocytes. *Cell.* 2007;131(6):1072–83. 10.1016/j.cell.2007.10.049 18083098

[101] Collins CR Hackett F Strath M: Malaria parasite cGMP-dependent protein kinase regulates blood stage merozoite secretory organelle discharge and egress. *PLoS Pathog.* 2013;9(5):e1003344. 10.1371/journal.ppat.1003344 23675297 PMC3649973

[102] Withers-Martinez C Strath M Hackett F: The malaria parasite egress protease SUB1 is a calcium-dependent redox switch subtilisin. *Nat Commun.* 2014;5: 3726. 10.1038/ncomms4726 24785947 PMC4024747

[103] Das S Hertrich N Perrin AJ: Processing of *Plasmodium falciparum* Merozoite Surface Protein MSP1 Activates a Spectrin-Binding Function Enabling Parasite Egress from RBCs. *Cell Host Microbe.* 2015;18(4):433–44. 10.1016/j.chom.2015.09.007 26468747 PMC4608996

[104] Hale VL Watermeyer JM Hackett F: Parasitophorous vacuole poration precedes its rupture and rapid host erythrocyte cytoskeleton collapse in *Plasmodium falciparum* egress. *Proc Natl Acad Sci U S A.* 2017;114(13):3439–44. 10.1073/pnas.1619441114 28292906 PMC5380091

[105] Koussis K Withers-Martinez C Yeoh S: A multifunctional serine protease primes the malaria parasite for red blood cell invasion. *EMBO J.* 2009;28(6):725–35. 10.1038/emboj.2009.22 19214190 PMC2647770

[106] Silmon de Monerri NC Flynn HR Campos MG: Global identification of multiple substrates for *Plasmodium falciparum* SUB1, an essential malarial processing protease. *Infect Immun.* 2011;79(3):1086–97. 10.1128/IAI.00902-10 21220481 PMC3067499

[107] Collins CR Hackett F Atid J: The *Plasmodium falciparum* pseudoprotease SERA5 regulates the kinetics and efficiency of malaria parasite egress from host erythrocytes. *PLoS Pathog.* 2017;13(7):e1006453. 10.1371/journal.ppat.1006453 28683142 PMC5500368

[108] Ruecker A Shea M Hackett F: Proteolytic activation of the essential parasitophorous vacuole cysteine protease SERA6 accompanies malaria parasite egress from its host erythrocyte. *J Biol Chem.* 2012;287(45):37949–63. 10.1074/jbc.M112.400820 22984267 PMC3488066

[109] Miller SK Good RT Drew DR: A subset of *Plasmodium falciparum* SERA genes are expressed and appear to play an important role in the erythrocytic cycle. *J Biol Chem.* 2002;277(49):47524–32. 10.1074/jbc.M206974200 12228245

[110] Thomas JA Collins CR Das S: Development and Application of a Simple Plaque Assay for the Human Malaria Parasite *Plasmodium falciparum*. *PLoS One.* 2016;11(6):e0157873. 10.1371/journal.pone.0157873 27332706 PMC4917082

